# A phase I study of extended dosing with lomeguatrib with temozolomide in patients with advanced melanoma

**DOI:** 10.1038/sj.bjc.6605016

**Published:** 2009-03-31

**Authors:** R F Kefford, N P B Thomas, P G Corrie, C Palmer, E Abdi, D Kotasek, J Beith, M Ranson, P Mortimer, A J Watson, G P Margison, M R Middleton

**Affiliations:** 1 Department of Medicine, Westmead Hospital, Westmead, New South Wales 2145, Australia; 2 Department of Medical Oncology, Churchill Hospital, University of Oxford, Old Road, Oxford OX3 7LJ, UK; 3 Oncology Centre, Addenbrooke's Hospital, Hills Road, Cambridge CB2 2QQ, UK; 4 Medical Oncology Unit, Tweed Hospital, Powell Street, Tweed Heads, New South Wales 2485, Australia; 5 Ashford Cancer Centre, 55 Anzac Highway, Ashford, South Australia 5035, Australia; 6 Sydney Melanoma Unit, Royal Prince Alfred Hospital, Missenden Road, Camperdown, New South Wales 2050, Australia; 7 Department of Medical Oncology, Christie Hospital, Wilmslow Road, Manchester M20 4BX, UK; 8 Kudos Pharmaceuticals, 410 Cambridge Science Park, Milton Road, Cambridge CB4 0PE, UK; 9 Cancer Research UK Carcinogenesis Group, Paterson Institute for Cancer Research, Wilmslow Road, Manchester M20 9BX, UK

**Keywords:** *O*^6^-methylguanine-DNA methyltransferase, lomeguatrib, temozolomide, melanoma

## Abstract

Lomeguatrib, an *O*^6^-methylguanine-DNA methyltransferase inactivator, was evaluated in an extended dosing regimen with temozolomide, designed according to pharmacodynamic data from previous studies. Patients with unresectable stage 3 or 4 cutaneous or unknown primary melanoma metastases were treated with lomeguatrib 40 mg, b.i.d. for 10 or 14 days and temozolomide 75–100 mg m^−2^ on days 1–5. Drugs were administered orally with cycles repeated every 28 days, for up to six cycles. A total of 32 patients were recruited to the study. Lomeguatrib for 10 days with temozolomide 75 mg m^−2^ was established as the optimal extended lomeguatrib dosing schedule, with haematological toxicity being dose limiting. There were two partial responses to treatment giving an overall response rate of 6.25%. Extending lomeguatrib administration beyond that of temozolomide requires a reduced dose of the latter agent. Only limited clinical activity was seen, suggesting no advantage for this regimen over conventional temozolomide administration in the treatment of melanoma.

Treatment for metastatic melanoma is unsatisfactory: the standard of care, dacarbazine, offers modest response rates and has never been shown to improve patient survival compared with purely supportive care. Despite these shortcomings and over 30 years of randomised phase 3 trials no other agent, or combination of agents, has supplanted dacarbazine (Atkins, 1997; Balch *et al*, 1997).

Much effort has been devoted to understanding the intrinsic and acquired resistance of melanoma to chemotherapy. The most commonly applied treatments, temozolomide and dacarbazine, are methylating agents that share the active moiety methyltriazineimidazolecarboxamide. Their cytotoxicity is mediated principally through methylation of DNA at the *O*^6^ position of guanine (Ockey *et al*, 1986; Margison and O'Connor, 1990; Kaina *et al*, 1997). Repair of the lesion by *O*^6^-methylguanine-DNA methyltransferase (MGMT) and/or tolerance based on deficiencies in mismatch repair or the engagement of apoptosis have been identified as important aspects of resistance to dacarbazine and temozolomide (Branch *et al*, 1993; Friedman *et al*, 1998). New molecules and experimental approaches have followed but, as yet, none has proved superior to single agent therapy.

Tumour cells frequently express high levels of MGMT, and pre-clinical studies identify the protein as a key determinant of cell survival (Chen *et al*, 1992; Kaina *et al*, 1997). Inactivation of MGMT before dosing with an *O*^6^-alkylating agent considerably enhances the anti-tumour activity of the latter drug *in vitro* and in animal tumour models (Dolan *et al*, 1990; Karran and Bignami, 1994; Friedman *et al*, 2002). We have previously reported the development of lomeguatrib, a small molecule inactivator of MGMT, and its use in combination with temozolomide. In a phase 1 trial we established lomeguatrib 40 mg per day and temozolomide 125 mg m^−2^ per day p.o. for 5 consecutive days as well tolerated (Ranson *et al*, 2006). Total depletion of MGMT occurred in three of five subcutaneous melanoma metastases analysed 4 h after the first dose of lomeguatrib, and >96% depletion was observed in the other two.

A randomised phase 2 study failed to demonstrate any improved efficacy for lomeguatrib/temozolomide, nor any responses in patients treated with the combination after progression on temozolomide alone (Ranson *et al*, 2007). Melanoma biopsies taken in the 24–72 h after patients finished their first cycle of lomeguatrib and temozolomide showed residual MGMT activity. The daily dose of lomeguatrib was increased to 80 mg, which remained well tolerated. It transpired that there was rapid recovery of MGMT levels once dosing with lomeguatrib was completed, allowing the repair of *O*^6^-meG before the two rounds of DNA replication required for cytotoxicity could occur. The pharmacodynamic data suggested that the inactivator should be administered for several days after temozolomide treatment, and the current study was devised to determine the tolerability of the chemotherapy regimen when given with 10 or 14 days of lomeguatrib. The MGMT inactivator was given at 80 mg per day (40 mg, b.i.d.) as this had been well tolerated with temozolomide in the randomised phase 2 trial. In addition, tumour response rates, pharmacodynamics (reported in the accompanying paper), time to progression and survival were investigated.

## Patients and methods

### Inclusion and exclusion criteria

Patients with histologically proven cutaneous melanoma or unknown primary melanoma with metastases were eligible for the study, provided that they had not previously received systemic chemotherapy for melanoma. Other requirements included stage III or IV measurable disease, age >18 years, Eastern Cooperative Oncology Group performance status of 0 or 1, life expectancy >12 weeks, adequate bone marrow and biochemical function (haemoglobin >10 g per100 ml, white blood cells >3 × 10^9^ per litre, absolute neutrophil count >1.5 × 10^9^ per litre, platelets>100 000 per *μ*l), creatinine ⩽1.25 upper limit of normal (ULN), bilirubin ⩽1.25 ULN, AST ⩽5 (metastases to liver) or 2 × ULN.

Patients were excluded if within 4 weeks of radio- or immunotherapy, pregnant or nursing, still recovering from surgery, suffering from significant comorbidity, had known brain metastases, had a history of seizures or were on anti-epileptic medication.

The study was conducted in accordance with the principles of the International Conference on Harmonisation of Good Clinical Practice guidelines and the Declaration of Helsinki Principles. The trial was approved by an independent ethics committee according to national and local requirements at each trial centre. All patients gave informed, written consent.

### Study design and statistical considerations

The trial was a multi-centre, dose escalation study in which approximately 40 patients were to be enrolled. The aim of the study was to define the optimally tolerated dose of temozolomide given for 5 days with lomeguatrib given for 10 or 14 days. If one of three patients at a dose level developed dose-limiting toxicity, up to three additional patients were treated at that dose level. If one of the three additional patients developed a dose-limiting toxicity, dose escalation ceased and six patients were treated at the preceding dose level. This lower dose level was defined as the maximum tolerated dose unless ⩾2 of 6 patients developed dose-limiting toxicity. Dose-limiting toxicity was defined as any of the following events: grade 4 neutropaenia lasting >5 days or if associated with infection/fever, grade 4 thrombocytopaenia for >5 days, grade 3 or 4 non-haematological toxicity (excluding grade 3 nausea and vomiting in patients who had not received optimal treatment with anti-emetics) and drug-related death. Expansion at the optimally tolerated dose was mandated to allow a preliminary assessment of efficacy, toxicity, pharmacokinetic and pharmacodynamic end points. A second expansion, to 80 patients, was permitted should the effects of the optimally tolerated dose warrant further study. The median time to progression and median survival time were estimated using Kaplan–Meier survival curves. Patients who had not progressed by the end of the study or who withdrew before progression and patients who had not died were censored for the respective analyses.

### Treatment

Lomeguatrib enteric-coated 10 mg capsules were obtained from Kudos Pharmaceuticals (Cambridge, UK), and temozolomide was purchased from Schering Plough Ltd (Welwyn Garden City, UK), as 5, 20, 100 and 250 mg capsules. Patients received lomeguatrib 40 mg, b.i.d. (p.o.) for 10 or 14 consecutive days every 4 weeks for up to six cycles. Temozolomide was administered on days 1–5 at 100 mg m^−2^ per day (p.o.) 2 h after lomeguatrib, with a planned escalation to 125 mg m^−2^ per day according to tolerability. Patients fasted for 1 and 2 h before and after temozolomide and lomeguatrib respectively.

A treatment delay of up to 2 weeks was allowed for resolution of drug-related toxicity. Dose reductions in temozolomide were mandated in the event of grade 4 haematological toxicity, grade 3 toxicity lasting 7 or more days or any grade 3 or 4 non-haematological toxicity. These were in decrements of 25 or 50 mg m^−2^ per day according to the type of toxicity encountered. Patients could be withdrawn for excessive toxicity, progressive disease, serious violation of the study drug protocol or withdrawal of consent.

### Evaluation of response and toxicity

All eligible patients who received any part of the treatment were considered assessable for toxicity. Patients were assessed for adverse events at each attendance. Physical exam, performance status and vital signs were recorded at the beginning of each treatment cycle. Complete blood count was checked before treatment and on days 14, 21 and 28, with blood chemistry tested on days 1, 14 and 28. Patients who developed grade 4 bone marrow suppression were assessed every 1–2 days. Adverse events were graded according to the National Cancer Institute-Common Toxicity Criteria version 2.0. Tumour response was assessed every second cycle based on clinical and radiological findings in accordance with the RECIST criteria.

### Pharmacodynamics

In a subset of patients peripheral blood mononuclear cell (PBMC) samples were obtained before treatment, 4 h after the first drug dose and at the end of treatment (day 10 or 14 depending on schedule) during cycle 1. Samples were assayed for MGMT activity, and levels of *O*^6^- and N7-methylguanine in DNA. Venous blood (5–10 ml) was collected into tubes containing 100 *μ*l 0.5 M EDTA and stored on ice for maximum of 4 h before isolation of PBMC and analysis of MGMT (Watson and Margison, 2000).

## Results

A total of 32 patients were enrolled in the study between August 2005 and May 2006, with characteristics as summarised in Table 1. All patients were evaluable for toxicity, but only 28 for efficacy. Four patients did not complete two cycles of treatment: three due to toxicity, one by choice.

### Toxicity

Two patients died of causes other than melanoma while on treatment. In one instance the cause of death was unable to be ascertained by the study team, in the other the patient was admitted with a neutropaenic fever. Melanoma in the patient had progressed and the patient developed septic shock and multi-organ failure, classified as probably and possibly related to treatment respectively. A total of 86 cycles of treatment were administered, with 46 having to be delayed and seven individuals needing dose reductions. All the delays and dose adjustments were due to haematological toxicity.

In the first cohort, of 10 days lomeguatrib with temozolomide 100 mg m^−2^ per day, all three patients experienced significant myelotoxicity and required dose reductions and delays in treatment. Temozolomide 75 mg m^−2^ per day with 10 days lomeguatrib was better tolerated. A third cohort, with lomeguatrib dosing extended to 14 days was also recruited. Nine patients were treated in this last cohort, which was twice expanded to further assess feasibility beyond cycle 1 after proving just tolerable in the terms described in the protocol. Three patients experienced grade 4 neutropaenia and/or neutropaenic fever in cycle 1, and all but one required a delay in administering subsequent cycles. The second cohort was therefore expanded to allow a more accurate assessment of efficacy.

Although all patients had at least one treatment-emergent adverse event, therapy was well tolerated aside from the increased haematological toxicity noted in cohorts 1 and 3. All treatment-related serious adverse events were neutropaenic episodes associated with fever and/or infection. The most frequent non-haematological toxicities attributed to treatment are summarised in Table 2, and were in keeping with those described for temozolomide alone.

### Efficacy

Of the 32 patients treated, 28 were evaluable for response. There were two partial responses to treatment, one each in cohorts 1 and 2, giving an overall response rate of 6.25%. A further 10 patients had stable disease, but in only 4 cases did this extend beyond the second assessment at the end of cycle 4. Median time to progression was 62 days, and median overall survival was 264 days. In view of the lack of efficacy the second expansion phase of the trial was not performed.

### Pharmacodynamics

Peripheral blood mononuclear cell samples were obtained from 10 patients during the course of the study. These were analysed for levels of DNA damage, specifically *O*^6^- and N7-methylguanine, and for MGMT expression. Detailed results are presented in the accompanying paper.

## Discussion

Prolonged inactivation of MGMT in this patient group failed to improve upon the activity expected from temozolomide alone. The response rate of 6.25% with combination therapy is disappointing, being approximately half that seen in the phase 3 comparison of temozolomide and dacarbazine and our earlier phase 2 trial of lomeguatrib and temozolomide – trials with similar entry criteria to the current study (Middleton *et al*, 2000; Ranson *et al*, 2007). Results were very similar to those achievable with temozolomide alone: median progression free and overall survival were 62 and 264 days, compared with 58 and 241 days in the phase 3 study.

From the point of view of toxicity, the patients fared as expected. The combination of lomeguatrib and temozolomide was well tolerated: although haematological toxicity was more frequent it was readily managed. Non-haematological side effects were very similar to those seen for the methylating agent alone, with nausea and vomiting, constipation, headache and fatigue predominant (Middleton *et al*, 2000).

The rationale for the current study came from the phase 2 trial involving lomeguatrib and temozolomide, where we observed recovery of tumour MGMT activity within 24 h of completing treatment (Ranson *et al*, 2007). In that trial the efficacy of lomeguatrib and temozolomide was no better than that of the cytotoxin alone. *O*^6^-methylguanine is toxic only through replication and may be repaired effectively at any point beforehand, so one possible explanation was that the recovery of tumour MGMT levels occurred before successive rounds of replication could cause cell death (Ollila *et al*, 1998).

As measured in PBMCs, extended dosing with lomeguatrib successfully depleted MGMT for the duration of treatment, and resulted in the persistence of DNA damage. There was an evident impact on bone marrow progenitor cells, because continued suppression of MGMT activity required a reduction in the dose of temozolomide given over 5 days due to haematological toxicity. Used alone a total of 1000 g m^−2^ temozolomide is deliverable, but with 5 days lomeguatrib only 625 mg m^−2^ is possible. Extending the lomeguatrib dosing to 10 days requires a further reduction to 375 mg m^−2^ temozolomide over the 5 days, and this dose is barely tolerable with 14 days of the inactivator (Middleton *et al*, 2000; Ranson *et al*, 2006, 2007).

Despite the effects of the MGMT inactivator on enhancing bone marrow toxicity, we failed to see a similar effect on melanomas, suggesting that tolerance of damage and/or failure of downstream elements required for cytotoxicity have greater importance in melanoma cells. It seems likely that other survival pathways prevent increased DNA damage from killing melanoma cells. For example, several defects in the apoptotic machinery have been reported in melanoma, such as upregulation of bcl-2, or epigenetic silencing of DAP kinase and Apaf-1. Another possible explanation for the difference between tumour and bone marrow is that tumour doubling times are such that the extension of MGMT inactivation to 10 or 14 days is still not sufficient to allow the two rounds of replication required for cell killing. Indeed in one series of patients studied for consideration of pulmonary metastasectomy, median tumour doubling time was 66.9 days (Ollila *et al*, 1998).

Our results, and those of others using *O*^6^-benzylguanine (Gajewski *et al*, 2005), hold out little hope for MGMT inactivation in the effective treatment of melanoma. Despite the wealth of pre-clinical data to support MGMT's role in determining the outcome of methylating agent treatment in melanoma, evidence for the protein's importance in the clinic is inconclusive (Middleton *et al*, 1998; Ma *et al*, 2002; Middleton and Margison, 2003). More might be achievable with better targeting of inactivation to tumour, but such agents are not yet available clinically. It also remains to be seen whether inactivation might offer a benefit in other tumour types, such as glioblastoma, where temozolomide is an active agent and the importance of MGMT in determining chemoresistance has been established (Hegi *et al*, 2005). In conclusion, administration of an extended schedule of lomeguatrib with temozolomide confers no advantage over conventional temozolomide dosing in melanoma.

## Figures and Tables

**Table 1:** Patient characteristics by dose cohort

**Cohort**	**1**	**2**	**3**
Lomeguatrib dose	40 mg, b.i.d., for 10 days	40 mg, b.i.d., for 10 days	40 mg, b.i.d., for 14 days
Temozolomide dose	100 mg m^−2^ per day	75 mg m^−2^ per day	75 mg m^−2^ per day
Number of patients	3	20	9
Median age (range)	70 (53, 81)	56 (35, 78)	51 (37, 71)
Gender (M/F)	3/0	12/8	3/6
			
*Stage*			
3	0	2	1
M1a	0	1	3
M1b	2	5	0
M1c	1	12	5
Performance Status (0/1)	1/2	19/1	8/1

M=male; F=female.

**Table 2:** Most common treatment-related toxicities^a^

	**All adverse events**	**Grade 3/4 adverse events**			
		**Total**	**Cohort 1**	**Cohort 2**	**Cohort 3**
	***N*=32 (%)**	***N*=32 (%)**	***N*=3 (%)**	***N*=20 (%)**	***N*=9 (%)**
Thrombocytopaenia	18 (56)	11 (35)	3 (100)	10 (50)	4 (44)
Neutropaenia	19 (59)	17 (53)	3 (100)	7 (35)	1 (11)
Febrile neutropaenia	9 (28)	9 (28)	0	4 (20)	1 (11)
Anaemia	5 (16)	0			
					
Nausea	20 (63)	1 (3)	0	1 (5)	0
Vomiting	10 (31)	2 (6)	0	2 (10)	0
Constipation	14 (44)	0			
Diarrhoea	6 (19)	1 (3)	1 (33)	0	0
Fatigue	13 (41)	1 (3)	0	1 (5)	0
Cough	9 (28)	0			
Anorexia	9 (28)	0			
Headache	12 (38)	0			
Dizziness	8 (25)	0			
					
Treatment delayed	24 (75)		3 (100)	13 (65)	8 (89)

a Adverse events considered possibly, probably or highly probably related to treatment occurring in 15% or more patients in any study arm.
